# Toward waterborne protozoa detection using sensing technologies

**DOI:** 10.3389/fmicb.2023.1118164

**Published:** 2023-02-24

**Authors:** Sara Nemati, Farzaneh Shalileh, Hamed Mirjalali, Kobra Omidfar

**Affiliations:** ^1^Foodborne and Waterborne Diseases Research Center, Research Institute for Gastroenterology and Liver Diseases, Shahid Beheshti University of Medical Sciences, Tehran, Iran; ^2^Department of Life Science Engineering, Faculty of New Sciences and Technologies, University of Tehran, Tehran, Iran; ^3^Biosensor Research Center, Endocrinology and Metabolism Molecular–Cellular Sciences Institute, Tehran University of Medical Sciences, Tehran, Iran; ^4^Endocrinology and Metabolism Research Center, Endocrinology and Metabolism Research Institute, Tehran University of Medical Sciences, Tehran, Iran

**Keywords:** waterborne protozoa, point-of-care, biosensors, microfluidic, smartphone

## Abstract

Drought and limited sufficient water resources will be the main challenges for humankind during the coming years. The lack of water resources for washing, bathing, and drinking increases the use of contaminated water and the risk of waterborne diseases. A considerable number of waterborne outbreaks are due to protozoan parasites that may remain active/alive in harsh environmental conditions. Therefore, a regular monitoring program of water resources using sensitive techniques is needed to decrease the risk of waterborne outbreaks. Wellorganized point-of-care (POC) systems with enough sensitivity and specificity is the holy grail of research for monitoring platforms. In this review, we comprehensively gathered and discussed rapid, selective, and easy-to-use biosensor and nanobiosensor technologies, developed for the early detection of common waterborne protozoa.

## 1. Introduction

Healthy water resources are still one of the main challenges in most countries, particularly less developed regions in dry parts of the world (WHO/UNICEF, 2014; Andrade et al., 2018). The lack of sufficient and appropriate water resources increases the use of contaminated water sources and wastewater for drinking, washing, and irrigation. Accordingly, employing contaminated water not only directly increases the risk of contamination by waterborne pathogens but also enhances the risk of contamination of vegetables with waterborne pathogens, particularly in farmlands irrigated using wastewater (Karanis et al., 2006; Javanmard et al., 2019, 2020).

Waterborne diseases include a large group of illnesses with mild to severe symptoms, which are caused by a broad spectrum of pathogens including bacteria, fungi, parasites, and viruses (Kalyoussef and Feja, 2014). Parasites, both protozoa and helminths, are important group of foodborne and waterborne pathogens, which can be transmitted from wild and domestic animals and contaminated water resources to humans (Sharma and Mutharasan, 2013; Vasilescu and Marty, 2016; Pazoki et al., 2020). The transmission route of foodborne and waterborne protozoa (FWP) is mostly oral-fecal and infection occurs when cysts or oocysts of a parasite are unintentionally ingested by a host (Kalyoussef and Feja, 2014).

Waterborne protozoa are responsible for a considerable number of outbreaks in the world. Regarding a recently published systematic review, 86.7% of eastern African countries reported at least one waterborne parasite from 1953 to 2019 (Ngowi, 2020). However, there is no periodical monitoring strategy for the detection of waterborne parasites (particularly protozoa) in less-developed regions like African countries. In contrast, outbreak surveillance in Europe between 2000 and 2007 indicated the presence of 354 and 70 outbreaks, related to drinking water and bathing water, respectively, in which protozoa were responsible for 17 (4.7%) drinking water and 38 (54.3%) bathing water outbreaks (ENHIS, 2009). Moreover, it was estimated that ~7,150,000 (95% CrI 3,880,000–12,000,000; 21.3%) illnesses in the United States in 2014 were associated with waterborne agents, of which *Giardia lamblia* and *Cryptosporidium* spp., were responsible for 415,000 (95% CrI 140,000–816,000) and 322,000 (95% CrI 61,700–993,000) illnesses, respectively (Collier et al., 2021). In addition, more than 10% of the total waterborne outbreaks from 1991 to 2008 were attributed to parasitic agents, particularly protozoa (Karanis et al., 2006; Efstratiou et al., 2017). More recently, *Cryptosporidium* spp., *G. lamblia, Cyclospora cayetanensis, Toxoplasma gondii, Blastocystis* sp., *Entamoeba histolytica*, microsporidia, and *Naegleria fowleri* were reported as the main causative agents detected in 251 waterborne outbreaks from 2017 to 2020 (Ma et al., 2022). Interestingly, most waterborne outbreaks due to protozoa have been reported in developed countries, indicating the importance of periodical monitoring of water resources, as well as diagnostic capabilities (Ma et al., 2022).

Significant progress has been made in recent decades in developing portable, reusable, and effective miniaturized systems or point-of-care (POC) platforms. POC tests can be performed outside a clinical laboratory setting, at or near the site of patient care. In addition, along with climate change, particularly in recent years, the risk of transmission of infectious diseases and the number of areas, which were previously unaffected by specific infectious diseases, have increased (Mora et al., 2022). In contrast, global warming, as a coming challenge in the world, decreases access to healthy water resources and increases emerging water and food safety concerns (Duchenne-Moutien and Neetoo, 2021). In fact, drought due to climate changes increases seasonal water resources, which aggregates animals and human communities in a region, recycled water resources, groundwater, and even lagoon water, and therefore, the risk of transmission of potentially pathogenic microorganisms, particularly parasites, from animals to humans (Titcomb et al., 2021). Moreover, because of the presence of a resistant stage, cyst/oocyst/egg, in the life cycle of parasites, particularly protozoa, these microorganisms endure harsh conditions such as drought much more than other microorganisms. All of these reasons highlight the importance of protozoa infections and the development of POC techniques for the detection of these parasites in the future. Guidelines, commonly needed for establishing well-organized POC systems, are presented by the World Health Organization (WHO). These guidelines are identified as ASSURED, in which the abbreviation ASSURED stands for affordable, sensitive, specific, user-friendly, rapid, equipment-free, or minimal, and delivered to those who require them (Syedmoradi et al., 2017, 2021; Omidfar et al., 2020).

This study highlights the need for rapid, selective, and easy-to-use technology for the early detection of common waterborne parasitic pathogens. The purpose of the current review is to provide a comprehensive overview of conventional methods and emerging biosensors and nanobiosensors, with a focus on recent advances in smart-based devices.

## 2. A brief look at the significant waterborne parasites

Although a broad spectrum of parasites is reported from waterborne outbreaks, *Cryptosporidium* spp., *G. lamblia, T. gondii*, and *E. histolytica* are among the most frequently detected waterborne parasites in the world (Al-Shamiri et al., 2010; Robert-Gangneux and Dardé, 2012; Plutzer and Karanis, 2016; Sarkari et al., 2016; Ma et al., 2022). Nevertheless, microsporidia, *Blastocystis* sp., *Dientamoeba fragilis, Balantidium coli, C. cayetanensis*, and *Isospora belli* are the neglected waterborne parasites (Karanis et al., 2006; Plutzer and Karanis, 2016). Regarding the worldwide waterborne outbreaks reported by Baldursson and Karanis (2011), from 2004 to 2010, *Cryptosporidium* spp., and *G. lamblia* were the major causative agents in 60.3% (120) and 35.2% (70) of 199 outbreaks, respectively, while *T. gondii, C. cayetanensis, Acanthamoeba* spp., *E. histolytica*, and *Blastocystis* sp., were the other reported agents. Recently, an update on waterborne outbreaks due to parasites from 2017 to 2020 signified the high prevalence of *Cryptosporidium* spp., and *G. lamblia* as the major reported agents, followed by *D. fragilis, T. gondii, C. cayetanensis, Blastocystis* sp., *E. histolytica, N. fowleri*, and microsporidia (Ma et al., 2022).

### 2.1. *Cryptosporidium* spp.

*Cryptosporidium* spp. are apicomplexan protozoa, with several known species that infect humans and many other vertebrates, and are transmitted *via* the fecal-oral route through ingesting oocytes in contaminated food or water (Leitch and He, 2012; Gerace et al., 2019; Zahedi and Ryan, 2020). *Cryptosporidium* spp. can infect both immunocompetent and immunocompromised individuals, of which two species, *Cryptosporidium parvum* and *Cryptosporidium hominis*, are the most prevalent species in humans (Mmbaga and Houpt, 2017).

*Cryptosporidium* spp. are known as the main protozoa parasite reported from waterborne outbreaks (Zahedi and Ryan, 2020; Gururajan et al., 2021; Zahedi et al., 2021). A most recent study reported that, from 251 waterborne outbreaks with parasitic agents, *Cryptosporidium* was identified among 198 outbreaks (Ma et al., 2022). *C. parvum* is a zoonotic species, which is isolated from humans and a broad spectrum of animals; therefore, there is an increased risk of contamination of water resources not only by human feces but also by excreted oocysts from free-range animals (Fernández et al., 2021; Mohammad Rahimi et al., 2022). In addition, it was documented that routine wastewater treatment processes including sedimentation, activated sludge, chlorination, and filtrations are not enough to eliminate *Cryptosporidium* oocysts from water samples (Sroka et al., 2013; Ramo et al., 2017), which increases the concern for the contamination of downstream farmlands irrigated with treated wastewater (Javanmard et al., 2020).

### 2.2. *Giardia lamblia*

*G. lamblia* (also known as *Giardia intestinalis* and *Giardia duodenalis*) is an anaerobic-flagellated non-invasive protozoan, which can infect the small intestine and, in a few cases, the distal small bowel, cecum, stomach, and pancreas of humans and many other vertebrates (Einarsson et al., 2016; Bahramdoost et al., 2021). *G. lamblia* is more prevalent in children living in developing countries and is classified as FWP. This protozoan is transmitted *via* the fecal-oral route through ingestion of the cystic form in contaminated food or water (Bello et al., 2011).

*G. lamblia* is the second-most reported protozoa from waterborne outbreaks worldwide (Karanis et al., 2006; Efstratiou et al., 2017; Ma et al., 2022). Similar to *Cryptosporidium* spp., the main reason for the high prevalence of *G. lamblia* in waterborne outbreaks is the capability of this protozoan to remain viable during water treatment processes (Sroka et al., 2013; Ramo et al., 2017). Large waterborne outbreaks due to *G. lamblia* have been reported all over the world, particularly in developed countries. Nygård et al. (2006) documented a large outbreak of giardiasis among at least 1,300 cases in Norway that was linked to leakage of wastewater pipes and insufficient wastewater treatment. Recently, a large giardiasis outbreak related to tap water occurred in Bologna Province, Italy, and the presence of *G. lamblia* was documented in 228 stool samples (Resi et al., 2021). However, the number of reported outbreaks due to *G. lamblia* and *Cryptosporidium* spp., in developed countries is significantly higher than in less-developed regions, which could be due to the use of more sensitive detection technologies in developed countries (Ma et al., 2022).

### 2.3. *Toxoplasma gondii*

*T. gondii* is an obligate intracellular parasite, which infects most warm-blooded animals, and Felidae family members are its definitive hosts (Tenter et al., 2000; Mendez and Koshy, 2017). *T. gondii* is transmitted *via* several routes including vertical transmission (Kanková and Flegr, 2007; Robbins et al., 2012; Chaudhry et al., 2014), transfusion and needle stick (Foroutan-Rad et al., 2016), and fecal-oral route *via* eating or drinking oocyst-contaminated food and water (Hill and Dubey, 2002). However, *T. gondii* is considered a neglected waterborne protozoan (Baldursson and Karanis, 2011; Karanis et al., 2013; Plutzer and Karanis, 2016). Nevertheless, in an outbreak related to drinking water in the Champagne-Ardenne region, France, Villena et al. (2004) detected *T. gondii* DNA in 10/125 analyzed samples. Then, Aubert and Villena (2009) analyzed water samples, which were collected in 2001 in Champagne-Ardenne, France, and characterized *T. gondii* DNA in 37/482 environmental samples. These two studies suggested the high contamination of water samples with *T. gondii* in the studied region in France. The presence of *T. gondii* DNA in wastewater samples in Germany was as high as that reported in France. Accordingly, Gallas-Lindemann et al. (2013) scrutinized influent and effluent samples of wastewater in Germany and reported the presence of *T. gondii* DNA in 8/83 (9.6%) samples using loop-mediated isothermal amplification (LAMP). Microscopically, oocyst-like positive samples for *T. gondii* were also detected in environmental water samples collected in the Galápagos Islands, Ecuador, although molecular identification of the samples was not successful (Verant et al., 2014). *T. gondii* DNA was also identified using real-time PCR in 2/8 wastewater samples, together with *G. lamblia, E. coli, Entamoeba dispar, Entamoeba hartmanni, Blastocystis* sp., and *Acanthamoeba* spp. in Spain (Moreno-Mesonero et al., 2022). These studies, as well as a large outbreak due to the consumption of drinking water contaminated with *T. gondii*, which was reported in 2018 from Santa Maria, Brazil (Minuzzi et al., 2021), highlight the importance of waterborne toxoplasmosis and the neglected role of *T. gondii* in waterborne outbreaks due to insufficient detection techniques.

## 3. Available diagnostic techniques for the detection of waterborne parasites

Diagnosis plays a critical role in the discovery of new pathogens, monitoring and surveillance, the prediction of epidemics and pandemics due to emerging and re-emerging pathogens, and antibiotic resistance (Mohammad Rahimi et al., 2019). In recent decades, various methods have been developed for the diagnosis of intestinal parasites. However, conventional diagnostic methods are still employed for the detection of intestinal parasites, particularly in less-developed regions (Mohammad Rahimi et al., 2019). Accordingly, the detection of *G. lamblia, Cryptosporidium* spp. and *E. histolytica* is mainly based on the optical detection of cysts/oocysts and trophozoites of parasites using microscopy (Destura et al., 2015; Ricciardi and Ndao, 2015; Hooshyar et al., 2019), while the most common diagnostic method for *T. gondii* is immunoassay techniques (Elgun and Koltas, 2011; Rostami et al., 2018).

Despite the advantages of microscopic methods, there are some limitations such as the technique being time-consuming and optical skills of laboratory technicians (Laude et al., 2016; Sakamoto et al., 2018). In addition, due to the low number of FWP in a large volume of environmental samples like water, employing microscopic techniques is an important challenge. For example, the concentration of *G. lamblia* in water samples has been reported to be 0.01 to 100 cysts/L (WHO, 2014); thus, developing methods for the detection of 1 cyst/oocyst of *G. lamblia* and *Cryptosporidium* spp., in 10–100 L of water samples is desirable, particularly when we consider that *Cryptosporidium* spp., and *G. lamblia* are among the waterborne pathogens with a high priority and with infectivity dosage less than 10 oocysts/cysts (WHO, 2014). In contrast, the development of molecular techniques in recent decades has overcome the limitations of conventional methods and has provided more sensitivity and specificity for the detection of pathogens (Tavares et al., 2011).

Although progress has been made in molecular techniques, there are still some disadvantages and limitations that restrict the application of molecular techniques. Multiple steps including DNA or RNA extraction, primer design challenges, false results due to undesirable primer interactions, and expensive equipment are the common challenges facing molecular methods (Garibyan and Avashia, 2013; Khurana and Chaudhary, 2018). Regarding the abovementioned limitations of conventional and advanced molecular methods, designing high-efficiency and field-adopted diagnostic devices with a simple user interface and a rapid protocol is the main priority (Luka et al., 2019) (Table 1).

**Table 1:** Comparative evaluation of conventional methods for the detection of FWP.

**Technique**	**Detection principle**	**Advantages**	**Disadvantages**	**Time**
Microscopy techniques	Using microscopes to screen samples	• Low cost and high feasibility • Study the structure	• Low sensitivity • Requires highly trained and experienced technician • Need for sample concentration prior to screening	30–45 min to overnight staining
Culture technique	Cultivation of parasites in a specific medium to either keep parasites alive or increase their number	• Specificity up to 100% • Availability of isolate • Providing a system to assess vaccine research • Study the biochemistry, physiology, and metabolism of the pathogens • Study of drug resistance	• Low sensitivity • High probability of contamination • Requires specific cultivation conditions • Time-consuming	3–15 days
Immunological techniques (ELISA)	Based on a specific antigen-antibody interactions and antigen or antibody detection	• Low cost • Sensitivity and specificity in the range of 93–100% • Screening a large number of samples • Providing large amounts of data about every contact to studied agent	• Cross reactions • Not suitable for real time screening of an infection • Not suitable for detection of most of intestinal parasites	15 min−5 h
Molecular techniques (PCR- based techniques)	Detection of a specific region of a target gene	• High sensitivity and specificity • Fingerprinting • Phylogenetic analysis	• High cost and need for specific equipment and instruments • Need for sample treatment before tests such as DNA/RNA extraction • The negative role of inhibitors in the amplification process • Wrong estimation of an infection due to amplification of dead microorganisms	2–4 h

In recent years, advanced devices have been developed to overcome the limitations of available techniques. For example, the sensitivity of microscopic techniques is not high enough, and a well-trained technician is needed to reduce the possibility of a false report. In addition, the staining procedure may take much time; these reasons are challenges at the time of outbreaks. In contrast to microscopic methods, serological and molecular techniques are not labor intensive, provide high sensitivity and specificity, and do not need a microscopist. Nevertheless, serological methods may provide cross reaction, and are not suitable for screening a population at the time of outbreaks or most intestinal FWP. In addition, due to expensive equipment and instruments for molecular methods, and the need for a well-equipped laboratory, most molecular-based approaches are not suitable for investigation of an outbreak (Mohammad Rahimi et al., 2019; Mahdavi Abhari et al., 2023). Therefore, focus has been dramatically increased on biosensors, such as POC tests, which can provide enough sensitivity and specificity, without the need for a well-trained technician or a specific facility.

## 4. Biosensors: Development and types

**Figure 1:**
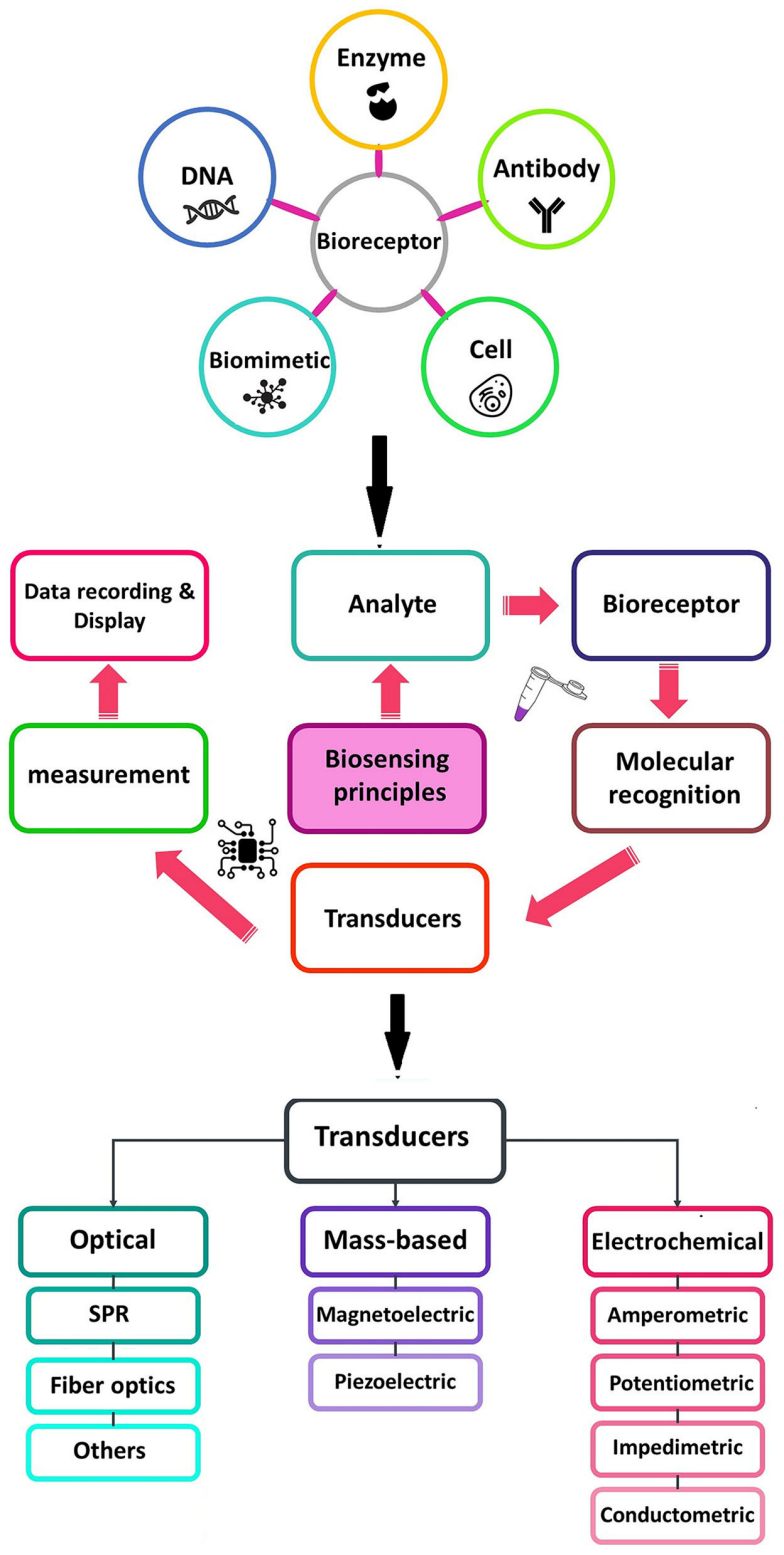
Schematic diagram of a typical biosensor consisting of various types of bioreceptors and transducers used in the biosensors.

The importance of food and water safety in various industries has led to the mining and improvement of nanoscale analytical devices known as nanobiosensors. Due to the numerous advantages of these devices such as portability, low cost, rapid assay time, ease of use, and high selectivity and sensitivity (Terry et al., 2005; Ahmadi et al., 2022; Khoshfetrat et al., 2022; Saeidi et al., 2022), particularly for the detection of infectious agents and pollutants in the environment (Pejcic et al., 2006; Sin et al., 2014; Ahmadi et al., 2021c), focus on the fabrication of biosensors as a diagnostic technology for the detection of different analytes in food, water, and environmental samples has intensely increased (Terry et al., 2005; Pejcic et al., 2006; Metkar and Girigoswami, 2018; Salouti and Khadivi derakhshan, 2020) (Figure 1).

A biosensor consists of at least two functional elements, namely, a molecular recognition element (receptor), which selectively interacts with its target analyte, and a physicochemical transducer. Biological elements are generally classified into enzymes, antibodies, and nucleic acids. A transducer is a component of biosensors which plays an important role in the signal detection process, and converts biological responses into a measurable signal with high quality (Khodaei et al., 2019; Ahmadi et al., 2021a,b).

Biosensors are generally classified into label-free and labeled. The latter biosensor employs labeled molecules for the detection of a target (Proll et al., 2007; Rhouati et al., 2016). Common labeling platforms are fluorescence or luminescence labeling, radiolabeling, isotope labeling, and enzymes (Deline and Nason, 2019; Ranjbar Bahadori et al., 2021). In these procedures, the final sensor signal represents the number of labels bound to target molecules. As a drawback, label-based technologies are labor- and cost-intensive and time-consuming (Cunningham and Laing, 2008). In addition, labeling of biomolecules can block active binding sites and alter the binding properties (Schöning and Poghossian, 2018).

In contrast, label-free biosensing technologies do not employ labels to facilitate measurements and instead incorporate the intrinsic physical properties of an analyte, such as the molecular weight, size, charge, electrical impedance, dielectric permittivity, and refractive index of a sample. In recent years, label-free biosensors have been developed due to their ability for rapid and inexpensive bio-detection in small reaction volumes (Schöning and Poghossian, 2018). Moreover, they can be integrated into lab-on-a-chip platforms, allowing monitoring of target analytes in real time. Label-free biosensors are usually designed based on optical, electrical or electrochemical, and acoustic parameters (Citartan et al., 2013).

Based on the biological elements, biosensors are categorized into genosensors, immunosensors, and aptasensors (Low et al., 2012; Kokkinos et al., 2016; Campuzano et al., 2017; Mohammed et al., 2017; Felix and Angnes, 2018).

**Figure 2:**
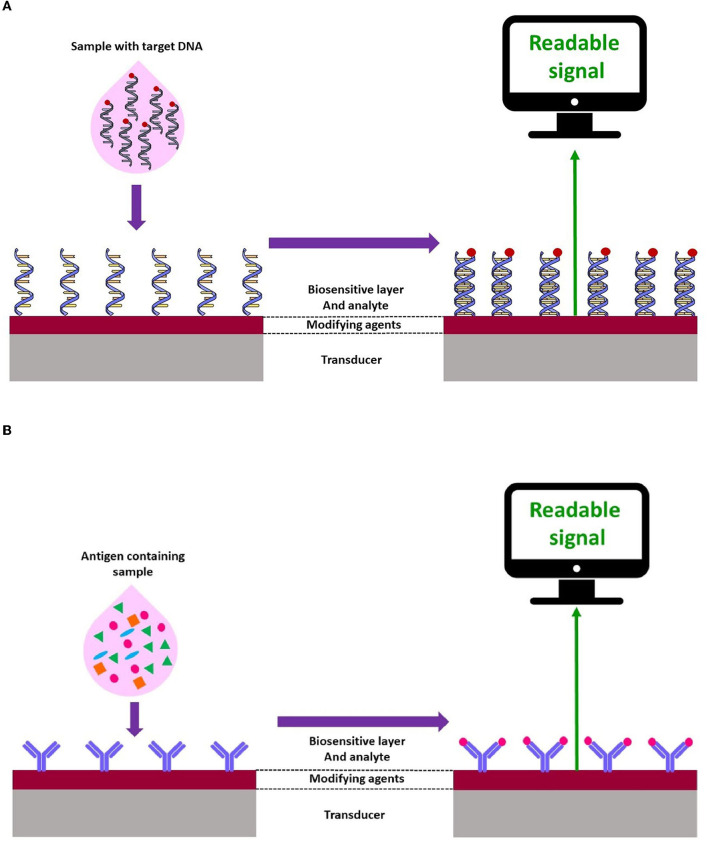
Schematic representation of electrochemical **(A)** genosensors and **(B)** immunosensor.

In genosensors, oligonucleotide sequences (DNA or RNA) are usually employed as bio-receptors, which are immobilized onto the transducer surface and hybridized with the single-stranded target DNA. In fact, the oligonucleotide sequences, such as a probe, recognize the analyte (sample DNA or RNA) by matching with the complementarity sequences. Genosensor-based devices are widely used for the detection of a broad spectrum of pathogens (Drummond et al., 2003; Babkina and Budnikov, 2006; Gao et al., 2010; Mohammed et al., 2017) (Figure 2A).

Immunosensors play an important role in the evaluation of specific elements in biological fluids. Currently, such assays have been extensively utilized in food safety and environmental analysis (Felix and Angnes, 2018; Hosu et al., 2018) (Figure 2B).

**Figure 3:**
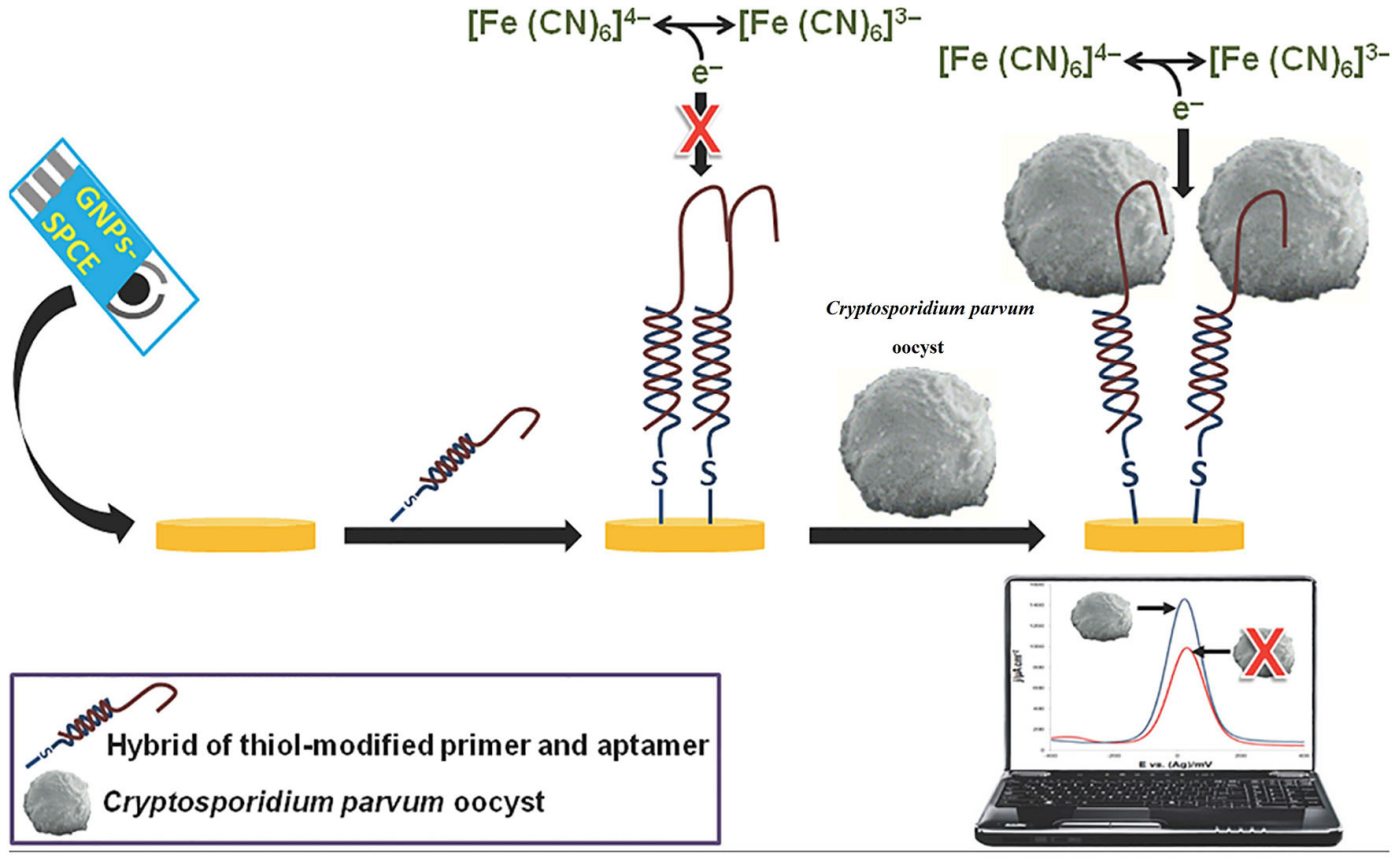
Illustration of a screen-printed aptamer-based electrochemical biosensing platform for the detection of *C. parvum* oocysts. Reproduced from Iqbal et al. (2015).

A single-stranded functional nucleic acid or peptide with a strong receptor property is recognized as an aptamer. Aptamers are usually constructed from combinatorial single-stranded libraries by the systematic evolution of ligands using the exponential enrichment (SELEX) method, and are applied to detect multiple target analytes (Shamah et al., 2008; Liu et al., 2020). Aptamer-based techniques have been applied for the detection of numerous pathogens such as human immunodeficiency virus (HIV), hepatitis B virus (HBV), hepatitis C virus (HCV), *Mycobacterium, Salmonella, Listeria, Staphylococcus, Clostridium, Bacillus, Escherichia, Aspergillus, Penicillium*, SARS-CoV, influenza virus, respiratory syncytial virus (RSV), *Trypanosome, Plasmodium, Cryptosporidium*, and *Leishmania* (Cho et al., 2011; Nagarkatti et al., 2012; Martín et al., 2013; Iqbal et al., 2015; Babamiri et al., 2018; Lavania et al., 2018; Li et al., 2018, 2020; Suh et al., 2018; Wei et al., 2018; Xi et al., 2018; Cai et al., 2019; Singh et al., 2019; Zou et al., 2019). The number of aptasensor-based studies for the detection of FWP is low. In this regard, Iqbal et al. (2015) developed an electrochemical nanomaterial-based aptasensor using a gold nanoparticle (NP)-modified screen-printed carbon electrode (SPCE) to detect *C. parvum* oocysts in spiked fresh fruits. In this system, 14 aptamer colons were discovered and anti-aptamer and thiolated DNA primers were mixed to produce a hybrid compound that was assembled onto the SPCE. The fabricated aptasensor recognized *C. parvum* with a wide dynamic range from 150 to 800 oocysts and a detection limit of ~100 oocysts. This study suggested promising findings for the detection of *C. parvum* in food products (Iqbal et al., 2015) (Table 2; Figure 3).

**Table 2:** Summary of selected biosensors for detection of various FWP.

**Analyte**	**Type of sensor**	**Transducer**	**Type of chip**	**Limit of detection**	**Range (linear range)**	**Source**	**References**
*G. lamblia*	Immunosensor	PEMC (piezoelectric excitation of millimeter cantilever)	PbZr_0.52_Ti_0.48_O_3_ (PZT) films and glass layer	1–10 cysts/mL per 15 min	0.5–5.0 mL/min/10–10,000 cysts/mL	Water	Xu and Mutharasan, 2010
*C. parvum*	Genosensor	Chronopotentiometric (electrochemical)	Screen-printed carbon strip electrodes (SPEs)	ng/mL levels of the *Cryptosporidium* DNA target	2.0 microgram/mL to ng/mL	Untreated drinking and river water	Wang et al., 1997
*C. parvum*	Immunosensor (labeled)	Evanescent wave fiber (optic chemical sensor)	RAPTOR Plus 4S	10^5^ oocysts/mL	10^6^/mL oocysts	Water	Kramer et al., 2007
*E. histolytica*	Immunosensor	Electrochemical sensor (voltammetry)	Gold screen-printed electrode	10 pg/mL (588 fM).	10 pg /mL (588 fM) to 500 pg/mL (29.4 pM)	Stool samples	Grewal et al., 2014
*C. parvum*	Immunosensor	Colorimetric detection (non-labeling fluorescence sensor)	Polydiacetylene-based fluorescence chip	1 × 10^3^ oocysts/mL	1 × 10^5^ oocysts/mL *C. parvum* (in tap water) and 1 × 10^5^ cysts/mL *G. lamblia* (in PBS buffer)	Water	Park et al., 2008
*C. parvum*	Immunosensor (label-free)	Electrochemical, FITc	Interdigitated gold electrodes (IDE)	40 cells/mm^2^	Between 15 and 153 cells/mm^2^	Water	Luka et al., 2019
*T. gondii*	Genosensor	Magnetic-fluorescent CdTe@Ni quantum dots (mQDs)	Not reported	2.70 × 10^−9^ mol/L	Not reported	*T. gondii* DNA	Xu et al., 2013
*T. gondii*	Immunosensor	Piezoelectric	Gold electrodes	1:5500	~1:5000–1:75	Infected rabbit serum	Wang et al., 2004
*T. gondii*	Genosensor	Magnetic fluorescent nanoparticles (Fe_3_O_4_/CdTe)	Not reported	8.339 x 10^−9^ M	ΔIF =1.805c + 10.804	*T. gondii* DNA	He et al., 2015
*C. parvum*	Immunosensor	Piezoelectric-excited millimeter-sized cantilever (PEMC)	PZT and glass film	100, 1000, and 10,000 oocysts/mL	100 to 10,000 oocysts/mL	Drinking water	Campbell and Mutharasan, 2006
*C. parvum*	Immunosensor	Electrochemical (potentiometric)	Screen printed electrode	5 × 10^2^ oocysts/mL	10^2^-10^6^ oocysts/mL	Fresh bovine feces	Laczka et al., 2013
*C. parvum*	Immunosensor	Optical surface plasmon resonance [SPR] biosensor	Gold chip	1 × 10^6^ oocysts/mL		*C. parvum* oocyst stock	Kang et al., 2006
*C. parvum*	Immunosensor	Piezoelectric electrochemical biosensor	Quartz crystal microbalance	3 × 10^5^-1 × 10^7^ oocysts/mL (~5 min).	3 × 10^5^-10^7^ oocysts/mL	Water	Poitras et al., 2009
*C. parvum*	Genosensor	Amperometric electrochemical biosensor	Interdigitated ultramicroelectrode array (IDUA) integrated with gold electrode	1 oocyst/mL	Not reported	*Cryptosporidium* oocysts DNA.	Nugen et al., 2009
*C. parvum*	Immunosensor (label-free)	Electrochemical impedance spectroscopy (EIS)	Biochip-based biosensing platform	< 10 cells/μL	1.43433 × 10^−5^ × C (oocysts/μl) + 7.545921 × 10^−4^	Water	Houssin et al., 2010
*C. parvum*	Immunosensor (label-free)	EIS electrochemical biosensor	Microfabricated gold electrode	20 cells/5 μL	Up to 200 cells/5 μL	Water	Luka et al., 2022
*C. parvum*	Aptasensor	Square wave voltammetry electrochemical sensor	Screen-printed carbon electrode	100 oocysts/mL	200–700 oocysts/mL	Fresh fruits	Iqbal et al., 2015

## 5. A brief look at nanomaterials incorporated into sensors

Improvements in nanotechnology science have provided the opportunity for researchers to work at nanoscale levels. Nanomaterials range from 1 to 100 nm and are classified into various groups, namely, nanoparticles (NPs), quantum dots (QDs), carbon nanotubes (CNTs), graphene, graphene oxide (GO), and nanochannels (Avant et al., 2019; Saleem and Zaidi, 2020; Pandey and Chusuei, 2021). Incorporating NPs with a biosensor system is performed for modifying and improving the sensor platforms and to overcome the limitations of conventional diagnosis tools (Luo et al., 2006). NPs are employed in the main types of biosensor systems including electrochemical, calorimetric, optical, and acoustic (Malik et al., 2013). NPs have critical functions such as reducing the time of reactions, catalysis, and immobilization of biomolecules (nucleic acid, antibody, and enzymes) into the electrochemical biosensors (Luo et al., 2006). In addition, due to their large specific surface, NPs are useful for improving electron transfer between biomolecules and the surface electrode (Cho et al., 2020). Common NPs include metal NPs (Au, Ag, and Pt), oxide NPs (SiO_2_, TiO_2_, ZrO_2_, and MnO_2_), and semiconductor NPs (CdS and PbS); metal NPs are more common due to their excellent catalytic properties in catalyzing electrochemical reactions (Tang et al., 2005). Moreover, silver and gold NPs have excellent conductivity properties, which enhance electron transfer between biomolecules and electrodes (Alaqad and Saleh, 2016). Moreover, gold NPs play an important role in increasing the sensitivity of electrochemical biosensors (Saha et al., 2012).

Magnetic NPs (MNPs) are used for designing magnetic biosensors, which have been broadly applied in medical areas such as diagnostic assays, DNA or RNA isolation, magnetic resonance imaging (MRI), and drug delivery (Khoo et al., 1997; Kudr et al., 2017; Wang et al., 2017; Ali et al., 2021). MNPs are a class of nanomaterials composed of metals such as cobalt, nickel, and iron, with paramagnetic, ferromagnetic, and superparamagnetic properties (Aboul-Enein et al., 1999; Akbarzadeh et al., 2012). Magnetic iron oxide NPs are employed for biomedical applications such as magnetic separation (Wu et al., 2015). For example, immunomagnetic separation (IMS) is now employed as a standard method for the detection and separation of *Cryptosporidium* spp., and *G. lamblia* oocysts/cysts from 10 to 150 L water samples (USEPA, 2012), although several studies have been conducted to increase the recovery rate of the method using either additional concentration methods or alternative elution (Hu et al., 2004; Fradette and Charette, 2022).

Oxide NPs have several chemical properties and possess a high surface energy (Stankic et al., 2016). For example, MnO_2_ NPs can directly react with biomolecules (Vukojević et al., 2018). In addition, oxide NPs, for instance, SiO_2_ NPs, can also be used as labels for biomolecules. SiO_2_ NPs, as an oligonucleotide label, have been used for electrochemical sensitive detection in genosensors and immunosensors (Ma et al., 2008; Wang et al., 2013).

Quantum dots are semiconductor nanocrystals made up of a reactive core, which contains semiconductor particles such as cadmium selenide (CdSe), cadmium telluride (CdTe), indium phosphide (InP), or zinc selenide (ZnSe). However, QDs, as ideal materials, have been widely used in the development of sensing technology due to their extraordinary chemical properties such as excellent optical aspects (Ding et al., 2022).

Carbon nanotubes are the most popular advanced sensing technology, and have recently attracted interest for their unique properties such as excellent electronic conductivity features and large surface-to-volume ratios (Zaporotskova et al., 2016). Nanotubes have cylindrical structures with several hexagonal graphite planes rolled in tubes, which are divided into single-walled NTs (SWNTs) and multi-walled NTs (MWNTs), based on the number of walls (Saxena and Srivastava, 2020).

Graphene and GO nanomaterials present unique chemical and electrical features, which have highlighted them as promising materials to improve signal responses in novel sensing technologies such as electrochemical biosensors, fluorescence resonance energy biosensors transfer (FRET), laser desorption/ionization mass spectrometry (LDI-MS), and surface-enhanced Raman spectroscopy (SERS) (Chauhan et al., 2017; Janegitz et al., 2017; Morales-Narváez et al., 2017). GO possesses a hydrophobic domain structure and hydrophilic oxygen-containing functional groups, which provide good biocompatibility and water dispersibility (Ghulam et al., 2022). However, their features, including high surface area and a high affinity for a variety of biomolecules (antibodies, enzymes, DNA, cells, and proteins), have made them ideal for next-generation biosensors (Lee et al., 2016).

The recent trends are the use of both single and array nanochannels for electrical biosensing applications. Graphene and its analogs are among the emerging materials used to obtain nanochannels (de la Escosura-Muñiz and Merkoçi, 2012). The applications of nanochannels are focused on the detection of DNA, protein, virus, toxin, and other analytes (Wang et al., 2015; Sun et al., 2016; Shiohara et al., 2022).

## 6. Applications of biosensors based on transducer types

The biosensor system employs a sensing technique and reacts with an analyte to produce a measurable electrochemical, electrical, mechanical, optical, or thermal signal (Mehrotra, 2016; Naresh and Lee, 2021). Biosensors can also be classified as electrochemical, optical or mechanical biosensors (Cammann, 1977; Thevenot et al., 1999; Ronkainen et al., 2010; Bermejo et al., 2011; Monosik et al., 2012; Ozdemir et al., 2013).

### 6.1. Electrochemical

In recent years, most studies on biosensors have focused on electrochemical systems (Ronkainen et al., 2010; Low et al., 2012; Kokkinos et al., 2016). The wide practical fabrication and usage of these biosensors are based on their advantages such as feasibility, portable, rapidness, low fabrication cost, simplicity of operation, and high selectivity of this system, which make these sensors quite desirable and attractive for the POC approach. Electrodes play an important role in the performance of electrochemical cells and biosensors. The electrode structure and properties influence the cost, sensitivity, selectivity, and limit of detection (LoD) of these biosensors (Faulkner and Bard, 2002; Cesewski and Johnson, 2020). In this regard, a label-free interdigitated-based capacitive biosensor was designed on interdigitated gold electrodes for the detection of *Cryptosporidium* oocysts in water samples (Luka et al., 2019). In this study, a capture probe, anti-*Cryptosporidium* monoclonal antibodies (IgG3) and bovine serum albumin (BSA), was employed to increase the specificity and to avoid non-specific interactions. The linear detection range for this technique was 15–153 oocysts/mm^2^ with a detection limit of 40 oocysts/mm^2^ (Luka et al., 2019).

Potentiometric biosensors have also been used for the detection of waterborne protozoa. Laczka et al. (2013) reported a novel electrochemical approach based on a potentiometric immunosensor for the rapid detection of *C. parvum* based on (HRP)*-*labeled secondary antibody, which was able to detect 5 × 10^2^ *Cryptosporidium* oocysts/mL in 60 min. In comparison to available ELISA techniques, Laczka et al. (2013) improved the LoD from 100- to 1,000-fold for the detection of oocysts, without the need for any specific antibody. In a study performed by Wang et al. (1997), a new electrochemical hybridization biosensor based on screen-printed carbon strip electrodes (SPEs) by the chronopotentiometry approach, as an electrochemical technique, was fabricated to detect a short specific nucleotide sequence of *Cryptosporidium* in untreated drinking and river water using the chronopotentiometric (CP) transduction method. This approach was able to discriminate *Cryptosporidium* DNA with an extremely low LoD, 50 ng/mL, and a short hybridization time of the probe, 3 min (Wang et al., 1997). Chronopotentiometry is a galvanostatic method that is used to study the mechanism and kinetics of chemical reactions with a constant level of current for a given period of time (Lingane and Peters, 1971; Kinyua Muthuri et al., 2021).

**Figure 4:**
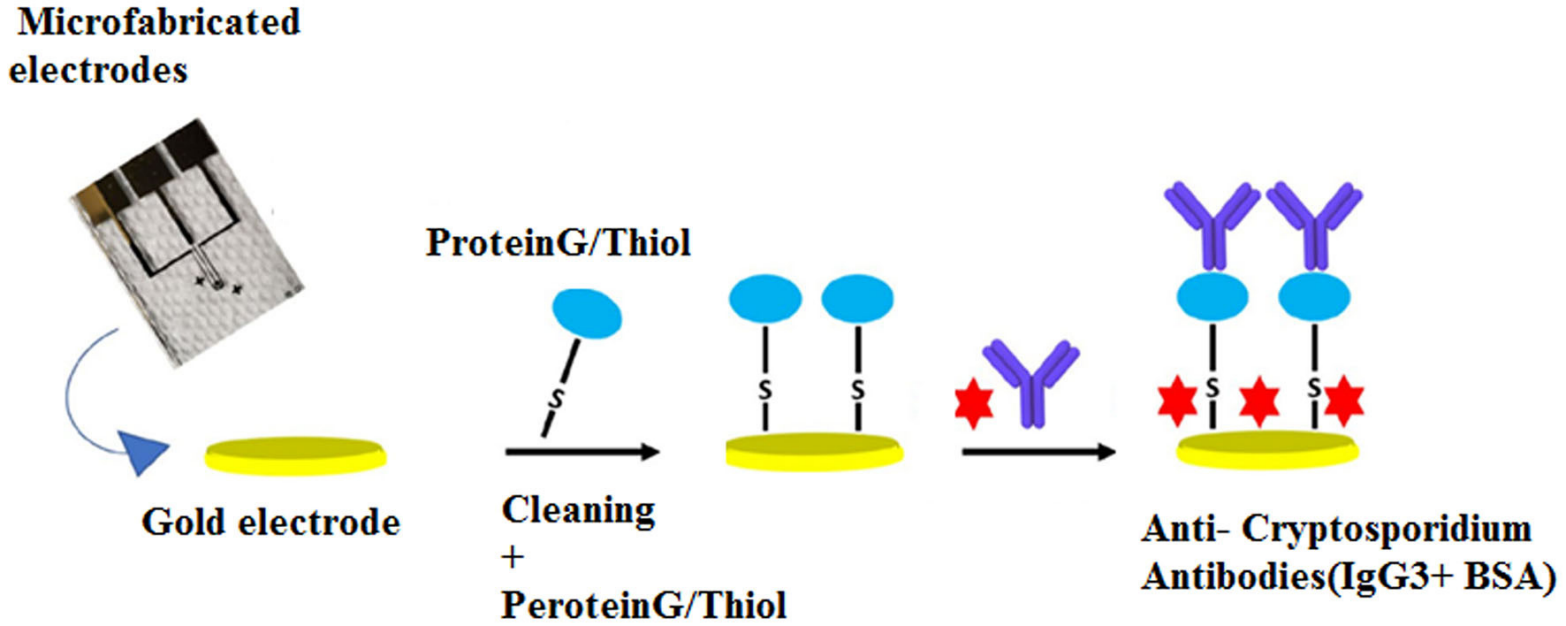
Chip-based device for label-free detection of *Cryptosporidium* oocysts in water. The figure shows the schematic process of the immobilization of anti-*Cryptosporidium* antibodies onto the Au electrode. Reproduced from Luka et al. (2022).

Electrochemical impedance spectroscopy (EIS) has been designed as a highly effective method based on label-free methods for the detection of biomolecules. It is used to investigate binding events that occur at the electrode surface (Magar et al., 2021). In the field of parasitology, Grewal et al. (2014) developed a nano-yeast-single-chain Fv (scFv) affinity reagents on a low-cost commercial gold screen-printed electrode for the sensitive detection of *E. histolytica* cyst antigens in stool samples at concentrations down to 10 pg/mL in buffer, with an inter-assay reproducibility of (% RSD, n = 3) 4.1%. A number of studies have also utilized this method for the detection of *Cryptosporidium* spp. A non-labeled detection system using a polydiacetylene (PDA)-based fluorescence chip based on a colorimetric detection system was developed for the detection of *C. parvum* with an LoD of 1 × 10^3^ oocysts/mL (Park et al., 2008). The main advantages of this study were real-time detection of *Cryptosporidium* spp. oocysts, rapidness, simplicity, and no need for any labeling or staining for analyses (Park et al., 2008). Houssin et al. (2010) fabricated a label-free EIS biochip-based biosensing platform for the detection of *Cryptosporidium* in water samples using EIS *via* an interdigitated microelectrode array with an LoD lower than 10 cells/μL. The authors suggested that this method could be proposed as an alternative technique to current staining procedures, which was able to differentiate live and dead oocysts based on electrical impedances between 10 kHz and 100 kHz (Houssin et al., 2010). More recently, Luka et al. (2022) reported a chip-based electrochemical biosensor for the sensitive and label-free detection of *Cryptosporidium* oocysts in water samples based on anti-*Cryptosporidium* monoclonal antibodies (IgG3). This novel platform was a fast, real-time, and inexpensive tool, which was utilized to measure *C. parvum* in the range of 0–300 oocysts, with an LoD of ~20 oocysts/5 μL (Figure 4).

### 6.2. Optical (fluorescence, chemiluminescence-based biosensor, and surface plasmon resonance [SPR]) biosensors

The main components of an optical diagnostic device are a light source, optical transmission medium (fiber, waveguide, etc.), immobilizedm biological recognition element (enzymes, antibodies, or microbes), and optical detection system (Chen and Wang, 2020). An optical sensor converts light rays into electronic signals *via* measuring the physical quantity of light and translating it into a readable signal (Deshmukh et al., 2020). Optical sensing technologies are also divided into label-based techniques such as fluorescent labeling and label-free methods (Tang et al., 2010; Bermejo et al., 2011).

Fiber optic biosensors (FOBs), as fluorescence-based optical biosensors, are increasingly being employed for the detection of foodborne and waterborne pathogens. This technique utilizes antibodies or other molecules to capture the target pathogen from a sample (Narayanaswamy, 2006; Hayman, 2008). In this regard, Kramer et al. (2007) developed an optical sensor (rapid automated FOB assay) based on a sandwich immunoassay using anti-*Cryptosporidium* oocyst polyclonal and monoclonal antibodies to detect the parasite in potable water. In this study, the polyclonal antibody captured the target pathogen and marked it with a cyanine 5-labeled (Cy5) detector monoclonal antibody. The LoD was 10^5^ oocysts/mL, while a 10-fold increase in sensitivity was achieved using the polyclonal antibody followed by boiling samples before the detection (Kramer et al., 2007); however, owing to the low infectivity dosage of *Cryptosporidium* spp., and the low concentration of oocysts in water samples, concentration and preparation steps before employing detection techniques are still required (WHO, 2014). To overcome the short lifetime of the excited state limitation of fluorescence (Berezin and Achilefu, 2010), a luminescence process, in which a photon may be released after any time, was developed (Gaft et al., 2015). Chemiluminescence-based biosensors are another type of optical sensing device (Aboul-Enein et al., 1999), which measure the rate of photon production and generate light through a chemical reaction (Kim et al., 2021). In this optical biosensor, the analyte interacts with the immobilized biomolecule, which is marked with chemiluminescence species. Some advantages of chemiluminescence tools are high sensitivity for the detection of pathogens, fast dynamic response, and a wide calibration range (Yan et al., 2021).

**Figure 5:**
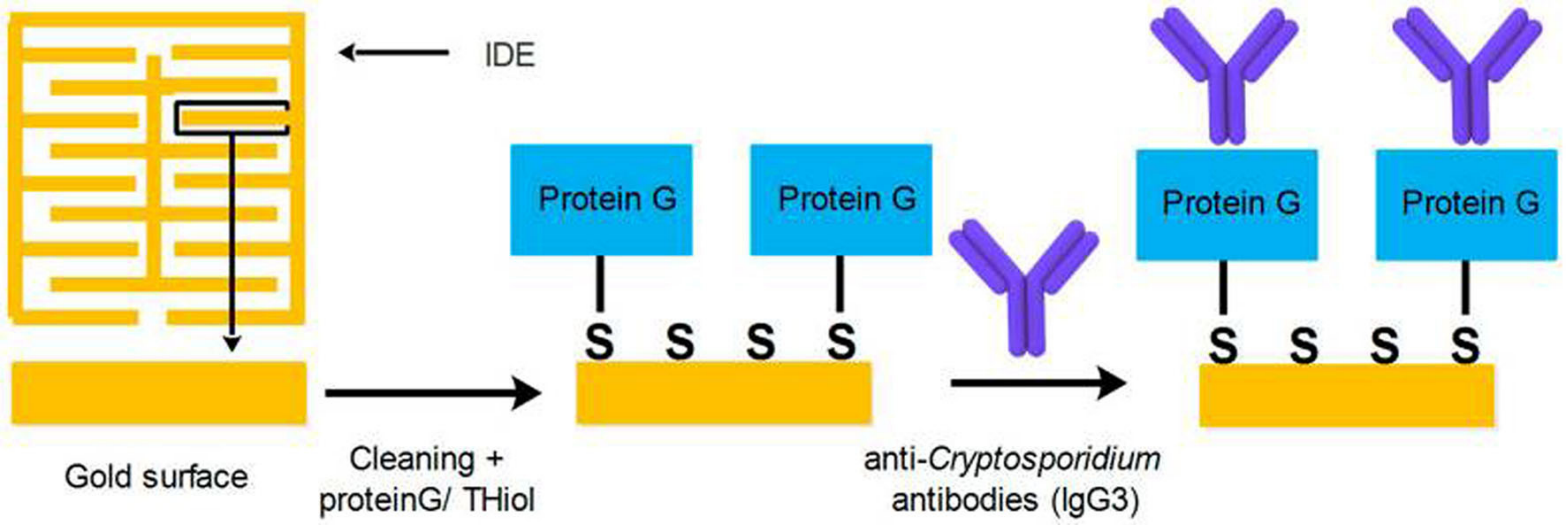
Procedure applied for covering the surface of IDE with SAM, and the attachment of anti-*Cryptosporidium* antibodies. Reproduced from Luka et al. (2019).

As a label-free-based biosensor, Luka et al. (2019) fabricated an interdigitated-based capacitive biosensor to detect *Cryptosporidium* oocysts in water samples. In this system, the number of *Cryptosporidium* oocysts captured on the surface of the electrode was identified by means of a fluorescein isothiocyanate (FITC) immunofluorescence assay. The result of this study indicated an LoD of 40 cells/mm^2^ and a linear range of detection between 15 and 153 cells/mm^2^ in environmental water samples. Briefly, anti-*Cryptosporidium* monoclonal antibodies (IgG3), as the capture biomolecules, were attached to the interdigitated gold electrodes (IDE) using the protein G/thiol. Finally, upon the formation of the *Cryptosporidium*-antibody complex, changes in the capacitive/dielectric properties were detected (Figure 5).

**Figure 6:**
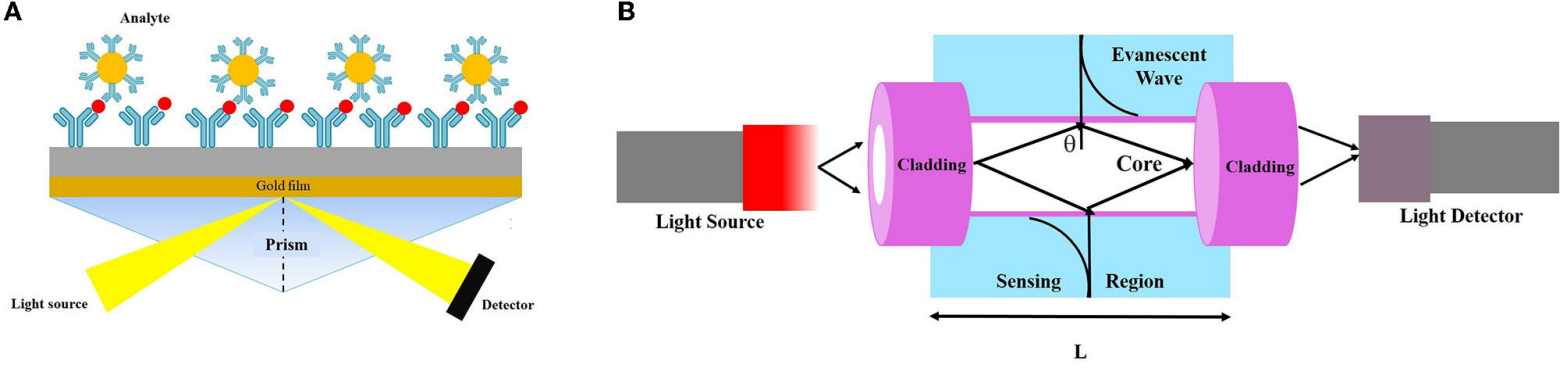
Schematic view of **(A)** evanescent wave fiber optic chemical sensor and **(B)** detection-based SPR technique.

SPR biosensors have been developed based on refractive index to increase the sensitivity of optical biosensors (Zeng et al., 2021). This system is a label-free optical phenomenon without radioactivity and fluorescence, which is recently considered a very powerful tool to study the interactions between the analyte and biorecognition molecules. This type of biosensor has remarkable advantages such as high sensitivity and specificity, label-free measurement, real-time analysis, and high-throughput capacity (Olaru et al., 2015). In addition to the common analytical applications, SPR devices are suitable for food safety monitoring and environmental applications (Olaru et al., 2015). In this regard, Kang et al. (2006) developed a flow-type SPR biosensor for the rapid detection of *Cryptosporidium* oocysts. Accordingly, an SPR biosensor was designed based on mixed self-assembled monolayers (SAMs) using 3-mercaptopropanol (3-MPOH) and 11-mercaptoundecanoic acid (11-MUA). These groups enhance the accessibility of analytes to the sensor surface using biotin–streptavidin biomolecules. This system was able to identify *C. parvum* oocysts in real time with an LoD of 1 × 10^6^ oocyst/mL, and the sensitivity was increased to ~1 × 10^2^ oocyst/mL using biotin–streptavidin biomolecules (Figure 6).

### 6.3. Mechanical biosensors

Mechanical biosensors are sensitive to alterations in mechanical characteristics. These types of assays play a critical role in different bioanalytical settings (Arlett et al., 2011; Zhang and Hoshino, 2019). The functions of mechanical biosensors are mostly based on either the induced stress on the cantilever platform or the alteration in the resonant frequency of a mass-spring device (Xu et al., 2019; Chalklen et al., 2020).

The piezoelectric system is a class of mass-based biosensors, which measures changes in the oscillating crystal resonance frequency due to the interaction between bioreceptor and biological elements (antibodies, enzymes, and antigens) (Nicu et al., 2005). Various piezoelectric (like quartz crystal) (Lim et al., 2020; Wu et al., 2021) and biosensing materials have been used in piezoelectric biosensors (Skládal, 2016).

Piezoelectric quartz crystal (PQC) immunosensors, as mass-sensitive devices, have been fabricated to calculate the quantification of biomolecular interactions (Bunde et al., 1998; O'Sullivan and Guilbault, 2000). Wang et al. (2004) developed a new, simple, rapid, and highly sensitive technique that was a promising alternative approach to detect anti-*T. gondii* antibodies (TgAbs) in clinical samples. The authors demonstrated that the latex piezoelectric immunoassay (LPEIA) was improved by using gold NPs, as an alternative to latex particles.

Another type of piezoelectric biosensor is a piezoelectric-excited millimeter-sized cantilever (PEMC) sensor that consists of a piezoelectric and a borosilicate glass layer with a sensing area (Zuehlke, 2022). A PEMC sensor was fabricated to detect the waterborne parasite, *G. lamblia*, in aquatic samples (Xu and Mutharasan, 2010). The resonant frequency of the sensor was continuously monitored using monoclonal antibodies against *G. lamblia* cysts, which were immobilized on PEMC sensors. In this procedure, 1–10,000 *G. lamblia* cysts/mL samples in a flow interacted with the antibody-immobilized sensor, and, upon binding cysts to the antibody, and changes in the resonant frequency of the cantilever sensor were continuously recorded. This method detected 10 cysts/mL for 15 min. Similarly, a PEMC biosensor was designed using immobilized IgM to detect *C. parvum* oocyst in a flow configuration at 1 mL/min. The PEMC detected *C. parvum* at 100, 1,000, and 10,000 oocysts/mL in less than 1 min. The resonance frequency response of the sensor was logarithmically correlated with the concentration of *C. parvum* oocysts, and due to the high sensitivity and specificity, it was employed for monitoring drinking water (Campbell and Mutharasan, 2008).

**Figure 7:**
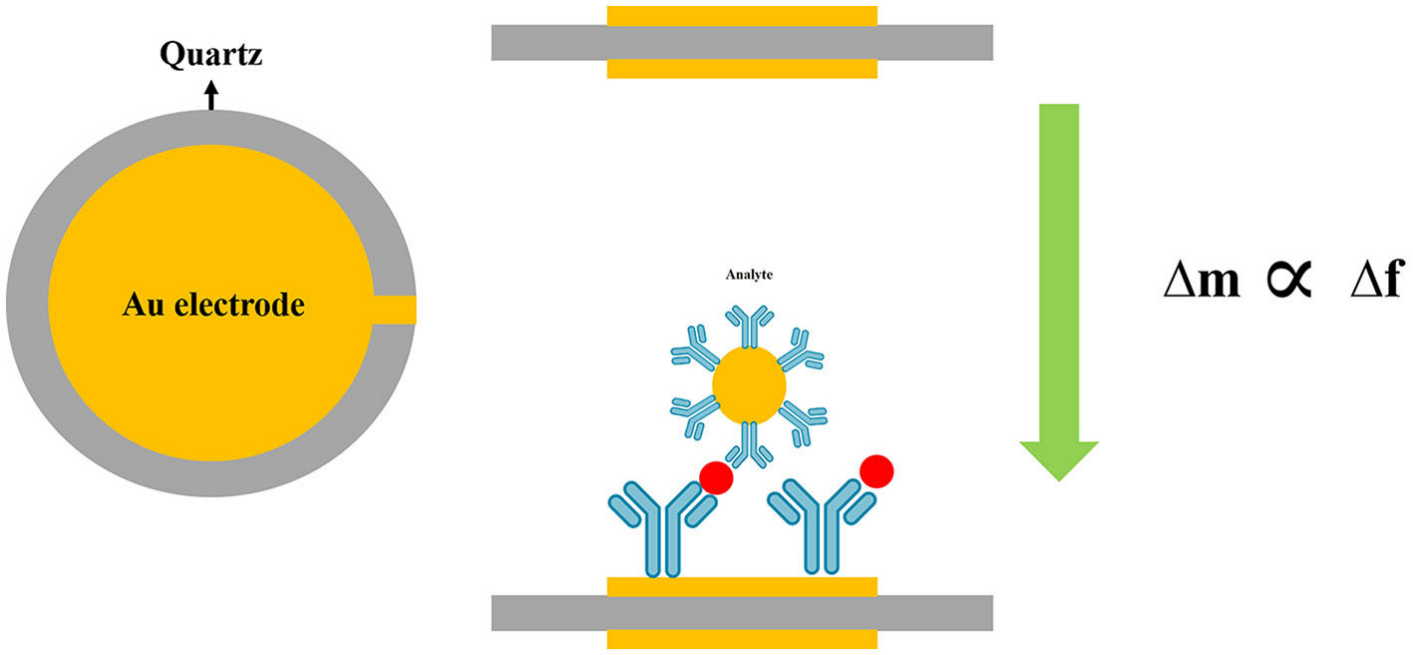
Schematic diagram of quartz crystal microbalance (QCM) technique.

A quartz crystal microbalance (QCM) with dissipation monitoring (QCM-D) was employed to detect *Cryptosporidium* oocysts in water samples (Poitras et al., 2009). Water samples are usually contaminated by a wide range of microorganisms, including bacteria, viruses, and parasites, that may cause interference during the detection of target pathogens in the biosensing system. To overcome this limitation, the QCM-D was used as a platform for the specific binding of *C. parvum* to an antibody-covered gold-coated crystal surface to increase the specificity of the method. This technique was able to detect oocyst concentrations from 3 × 10^5^ to 1 × 10^7^ per mL of water with a rapid operation (~5 min) (Poitras et al., 2009) (Figure 7).

MNP-based approaches are able to rapidly detect FWP with high sensitivity and selectivity (Akbarzadeh et al., 2012). MNPs have been intensively studied due to their capability to be employed in many areas such as magnetic storage devices, optical magnetic materials, magnetic separation, and DNA-targeted diagnosis (Duguet et al., 2006; Reddy et al., 2012). In the field of parasitology, in a study developed by Xu et al. (2013), magnetic-fluorescent CdTe@Ni quantum dots (mQDs) were utilized to design a sensitive nanobiosensor based on fluorescence resonance energy transfer (FRET) in order to detect *T. gondii* DNA. In this study, mQDs and commercial BHQ_2_ were the energy donors and acceptors, respectively. To produce a sensing probe, sCdTe@Ni mQDs and BHQ_2_ were used to label a stem-loop *T. gondii* DNA oligonucleotide at the 5′ and 3′ ends, respectively. This system was able to detect the target DNA of *T. gondii* with a LoD of ~2.70 × 10^−9^ mol/L. In addition, a study developed by He et al. (2015) detected *T. gondii* using the quenching of magnetic fluorescence NPs (Fe_3_O_4_/CdTe) based on CdTe QDs, which were synthesized using 3-mercaptopropionic (MPA) capping for *T. gondii* DNA detection, with a LoD of 8.339 × 10^−9^ M of DNA. In this study, similar to Xu et al. (2013), a stem-loop *T. gondii* DNA oligonucleotide was employed, which was conjugated to Fe_3_O_4_/CdTe at the 5′ end as the energy donor and BHQ_2_ at the 3′ end as the acceptor.

## 7. Microfluidic devices

Microfluidic systems are geometrically small scale (typically sub-millimeter) and can be incorporated with biosensor systems. Theoretically, microfluidic devices are comprised of thin grooves or small wells, channels, micro-channels, and chambers. These devices are rapid and accurate, and are increasingly employed for the detection of waterborne pathogens (Woolley and Mathies, 1994; Stone et al., 2004; Fiorini and Chiu, 2005; Chin et al., 2012).

In a biosensor-independent manner, there is a commercial microfluidic device for the detection of *Cryptosporidium* oocysts in water samples with on-chip integrated sample preparation features, named CryptoDetect CARD. This technology involves integrated immunomagnetic separation (IMS); however, the technology needs more development to specify the LoD and sensitivity, and the need for sample preparation, filtration, and concentration still limits its use (Rheonix, 2011).

**Figure 8:**
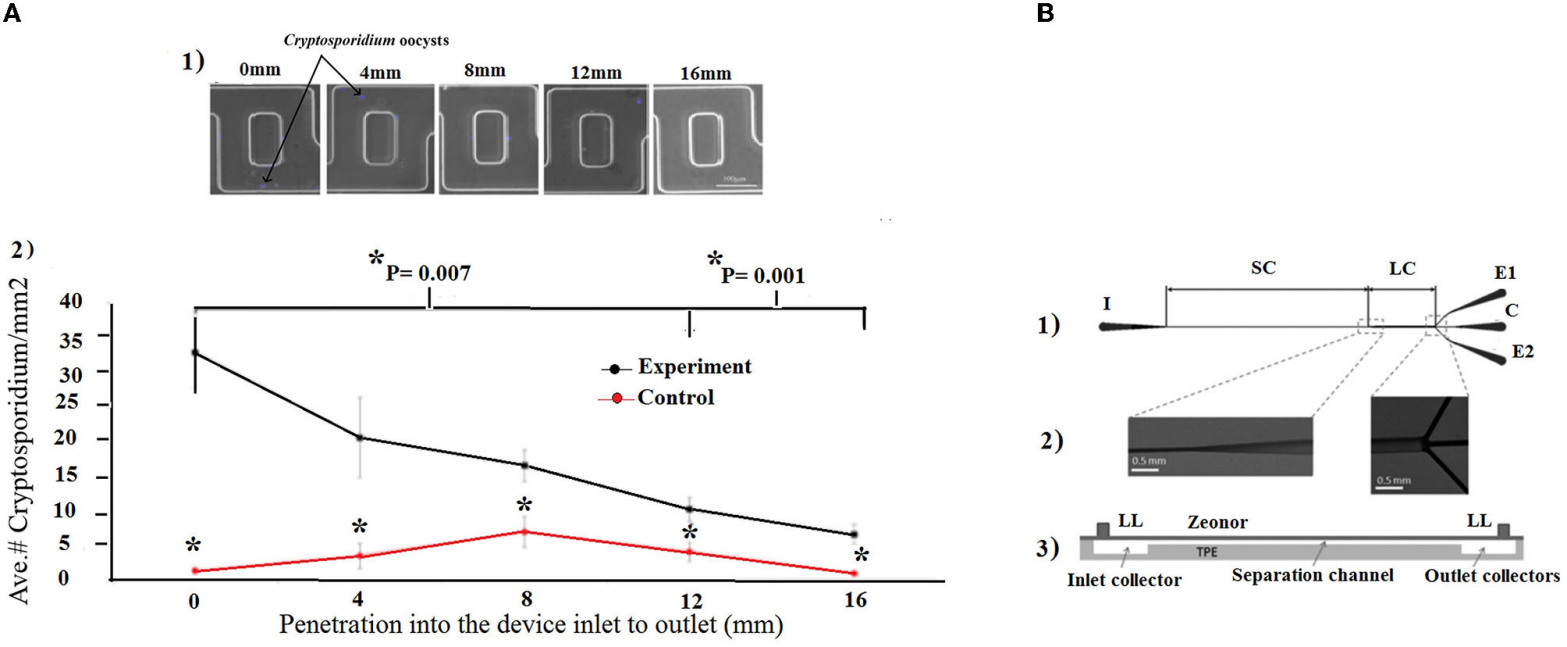
**(A)** Microfluidic micromixer device. (1) The captured oocysts at different lengths in the device were systematically counted randomly in 10 areas. (2) The number of oocysts per area was averaged at each length. ^*^indicates statistical significancy. Reprinted from Diéguez et al. (2018). **(B)** Microfluidic chip. (1) The inlet (I) port allows entry into the separation channel (SC), which then widens into a large channel (LC) that splits into three outlet collectors (C, E1, and E2). (2) Scanning electron microscopy images, and (3) schematic view of a vertical section, the Zeonor-TPE chip assembly with the Luer lock (LL) ports, separation channel, and input and output collectors. Reproduced from Ganz et al. (2015) with permission from the American Society of Microbiology (ASM) publication.

As a first strategy, hydrodynamic trapping together with immunofluorescence detection was utilized (Zhu et al., 2004; Taguchi et al., 2005, 2007; Lay et al., 2008; Mudanyali et al., 2010). In this regard, Taguchi et al. (2005) developed a micro-well array strategy to capture oocysts. For trapping oocysts in wells, micro-wells with a 10 μm or 30 μm diameter and a 10 μm depth were developed to capture oocysts of *Cryptosporidium*. This technology was able to detect the oocysts in very small sample volumes and could therefore be used instead of visual inspection of microscope slides. The micro-wells were coated with streptavidin and anti-*C. parvum* antibodies, and the samples containing *C. parvum* oocysts (10^7^ oocysts/mL) suspended in PBS were simply deposited onto the array. This approach was able to detect *Cryptosporidium* oocysts for 60 min at a maximum flow rate of 350 μL/min (5 mL could be processed in under 15 min), with a LoD of 36 oocysts/mL (Taguchi et al., 2005). Diéguez et al. (2018) purposed a disposable microfluidic micromixer, which was able to specifically capture, isolate, and concentrate *Cryptosporidium* from water samples. This designed device was able to analyze the quantification of captured oocysts using immunofluorescence microscopy, as well as an imaging flow cytometer. In addition, the microfluidic micromixer device provided a rapid and efficient detection method, with a capture efficiency of 96% compared to other available laboratory-scale technologies for the detection of *Cryptosporidium* oocysts (Figure 8A).

In addition, a microfluidic inertial separation chip was designed and fabricated for the separation of *Giardia* cysts from food samples (Ganz et al., 2015). The microfluidic chips consisted of an inlet, a main separation channel with a rectangular microfluidic channel, and a large channel, which was divided into three smaller channels connected to three output channels. The method was very efficient and specific for *G. lamblia*, and recovered an average of 68.4% of cysts, with a LoD of 38 cysts from a 25 g lettuce sample (Ganz et al., 2015) (Figure 8B).

**Figure 9:**
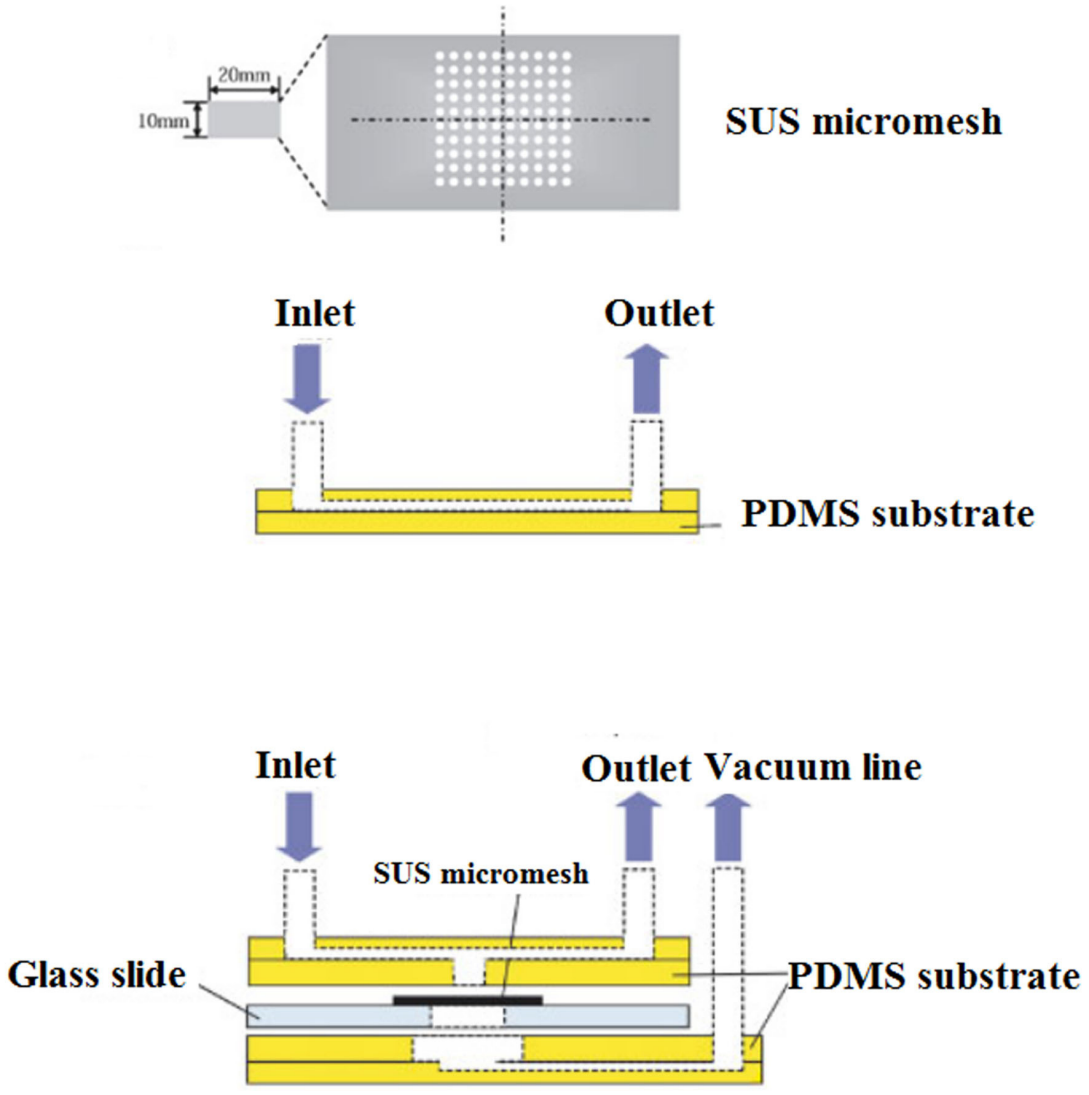
Schematic overview of the experimental setup of a micromesh microfluidic. (1) View of the SUS micromesh. (2) Side view of a PDMS microfluidic device equipped with the SUS micromesh, and (3) side view of a PDMS microfluidic device with microchannel, sample inlet, and outlet. Reproduced from Taguchi et al. (2007) with permission from Wiley & Sons.

Hydrodynamic trapping of *Cryptosporidium* oocysts, either in wells or filters, through pre-filter structures or a raindrop filter, was also developed. This microfluidic device was incorporated into a SUS micromesh to capture *C. parvum* oocysts. Trapped *C. parvum* oocysts were visualized by fluorescent staining. The concentration of added *C. parvum* oocysts and oocysts detected by the SUS micromesh was linearly correlated within the range of 18–200 oocysts/mL. The results of this technique were in agreement with the direct immunofluorescence assay coupled with the immunomagnetic separation (DFA-IMS) method, while the recovery of SUS micromesh (93%) was higher than DFA-IMS (90%), suggesting that the SUS micromesh is a promising procedure for counting *C. parvum* oocysts (Taguchi et al., 2007) (Figure 9).

As a second strategy, it was illustrated that trapping of *Cryptosporidium* oocysts using sieves or filters may increase the sensitivity of microfluidic systems (Zhu et al., 2004; Lay et al., 2008). A fully automated system consisting of a filtration unit and pumping system (1,000 L within 24 h), complemented by a microfluidic chip, Crypto-Tect bio-slide, was developed by the Shaw Water Ltd. Company, which stained and counted *Cryptosporidium* oocysts with a LoD slightly higher than 10 oocysts (Shaw, 2009), while 1,000 L drinking water was concentrated to a 1.5 mL sample, which was suitable for introducing to the microfluidic system. In addition, a microfluidic device based on the detection of *C. parvum* and *G. lamblia* oocysts/cysts using positive pressure was developed that identified *C. parvum* and *G. lamblia* fluorescent labels, while the staining solution was 10 to 100 times more diluted than the recommended concentration in the conventional glass method (Zhu et al., 2004).

**Figure 10:**
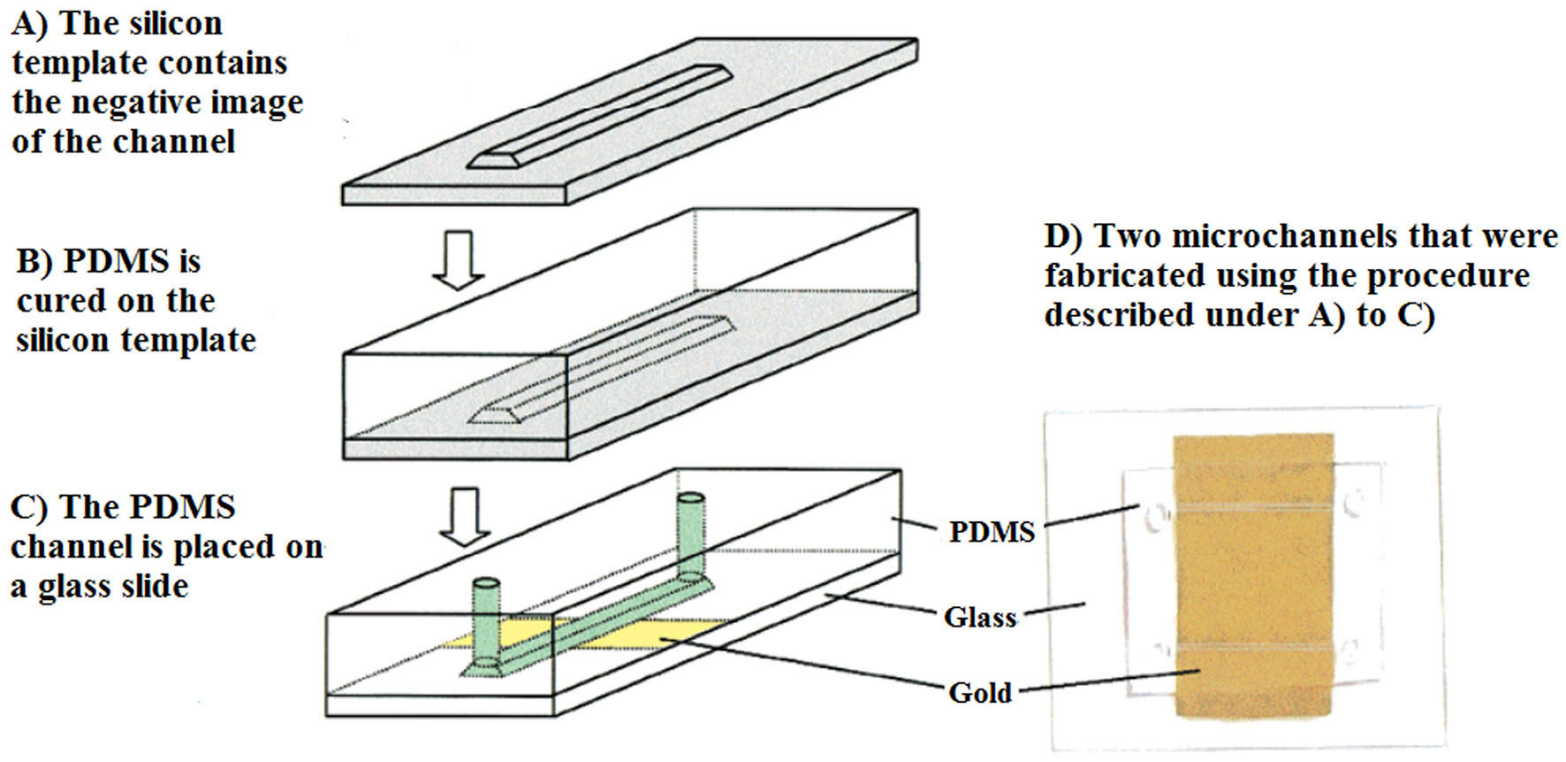
View of a PDMS microchannel. The channel was fabricated in silicon and placed on a glass slide, where gold was deposited. Reprinted with permission from Esch et al. (2001) copyright from the American Chemistry Society.

Microfluidic trapping devices can also be integrated with on-chip molecular methods for further applications (Mahdavi Abhari et al., 2023). Molecular sensing techniques include pre-amplification of the microorganism genomic material, either *via* fluorescence or electrochemical tools. These reliable and rapid detection techniques still require genomic materials. The detection of *C. parvum* in water resources still requires the parasite to be collected and concentrated from a large water sample volume. In fact, during the analysis of water samples, the numbers of recovered parasites are usually low and cannot be detected without DNA amplification. To overcome this limitation, Esch et al. (2001), developed a microfluidic chip, which was amplified by nucleic-acid-sequence-based amplification (NASBA), using DNA-modified liposomes to detect RNA in viable *C. parvum*. A NASBA-generated amplicon was placed between the capture and reporter probes in a microfluidic channel. To generate fluorescence, reporter probes were tagged with carboxyfluorescein-filled liposomes, which increased the sensitivity of detection, even in very low concentrations of targets. The LoD of the microfluidic chip was reported to be 5 fmol of amplicon in 12.5 μL of sample solution (Figure 10).

**Figure 11:**
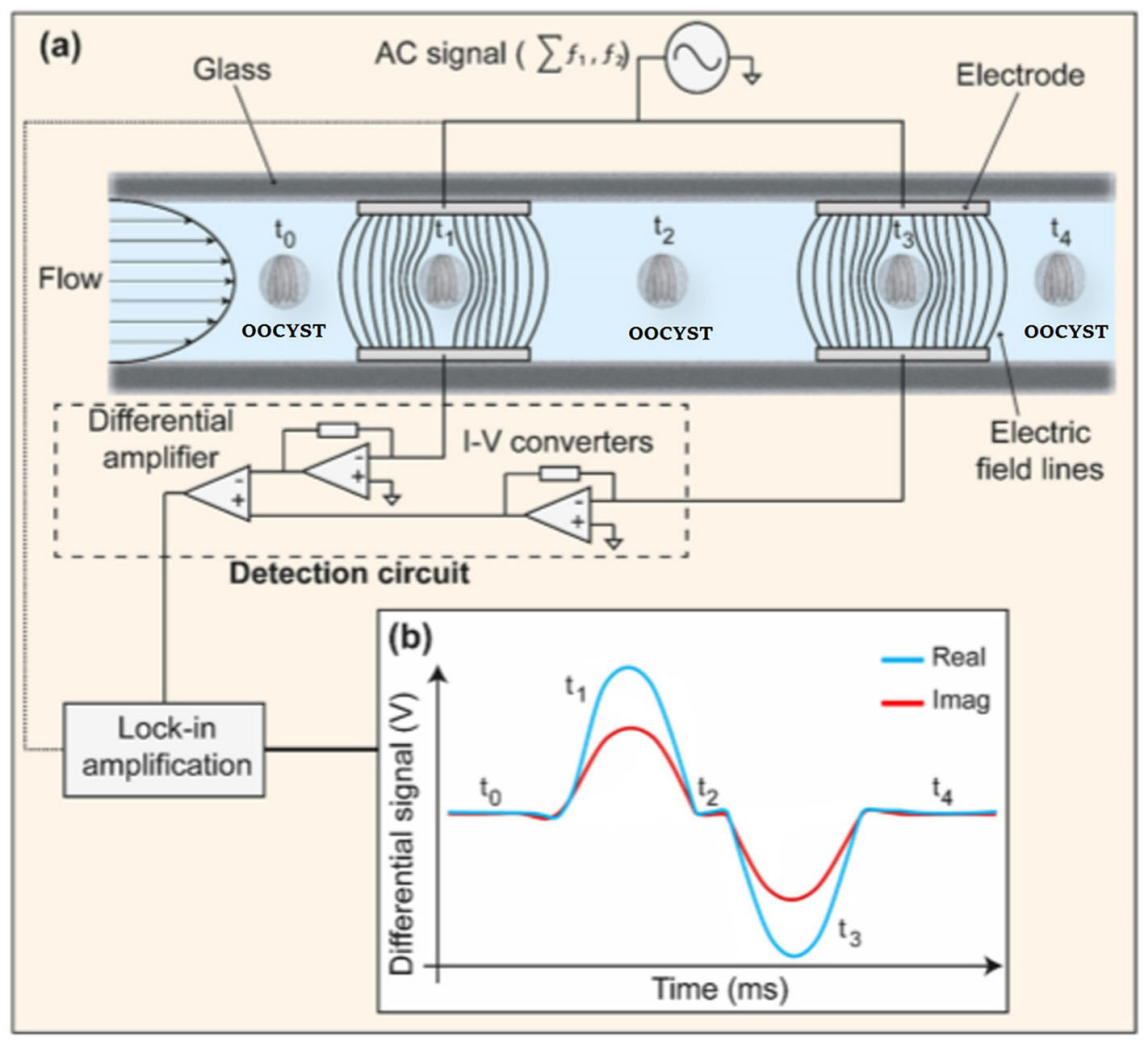
Schematic picture of a microfluidic impedance cytometer. **(a)** Two parallel facing electrodes. The electrodes were fabricated within a microfluidic channel. **(b)** The current flowing through the bottom electrodes was measured using a custom detection circuit. The circuit consists of trans-impedance amplifiers, which convert current (I) into voltage (V), and a differential amplifier. Reproduced from McGrath et al. (2017).

Recent developments have been presented in novel engineering systems for the detection of *Cryptosporidium* and *G. lamblia* based on the integration of electrochemical biosensors into microfluidic systems. A microfluidic impedance cytometry (MIC) system based on the detection of viable parasites was proposed and designed by McGrath et al. (2017), which was able to rapidly discriminate live and inactive *C. parvum* oocysts with over 90% certainty, and to identify the viability of *Cryptosporidium* and *Giardia* at the single (oo)cyst level (Figure 11; Table 3).

**Table 3:** Studies on microfluidic systems for the detection of FWP.

**Type of detection**	**Target**	**Limit of Detection (LoD)**	**Fabrication technique**	**Sample source**	**References**
Microfluidic device (micro-well array)	*C. parvum*	36 oocysts/mL in 60 min	Micro-well array strategy to trap and capture oocysts	Water	Taguchi et al., 2005
Microfluidic micromixer device	*C. parvum*	10 oocysts/L	Microfluidic micromixer to capture and isolate oocysts	Water samples	Diéguez et al., 2018
Microfluidic inertial separation chip	*Giardia*	38 cysts/mL	Microfluidic chips consists of a rectangular microfuidic channel	Food samples	Ganz et al., 2015
Microfluidic device (optical detection)	*C. parvum*	Not reported	Microfluidic device was incorporated with a SUS micromesh to trap oocysts	Water	Taguchi et al., 2007
Filter-based microfluidic device	*C. parvum* and *G. lamblia*	Not reported	Filter-based microfluidic device with immunofluorescent labeling to rapidly detect *C. parvum* and *G. lamblia*	Not reported	Zhu et al., 2004
Microfluidic chip	*C. parvum*	5 fmol of amplicon in 12.5 μL of sample solution	Detection of RNA, amplified by nucleic-acid-sequence-based amplification (NASBA) to detect viable *C. parvum*	Water	Esch et al., 2001
Microfluidic impedance cytometry (MIC) system	*C. parvum, C. muris* and *G. lamblia*	< 10 *C. parvum* oocysts/ μL	Detection of *Cryptosporidium* and *G. lamblia* based on integration of electrochemical biosensors into microfluidic systems	Not reported	McGrath et al., 2017
Optical microfluidic biosensors	*C. parvum*	1–10 oocysts/mL in 10 minutes	Microfluidic chip based on agglutination assay	Water	Angus et al., 2012

## 8. Smartphone microscopic method

In recent years, smartphone microscopic methods have been described and used as an alternative platform for the detection of targeted pathogens, incorporated with traditional optical microscopic methods. The strengths of these techniques are the low cost, small size, being portable, and easy transportation to rural and remote settings. In fact, portable devices that can transmit relevant data to remote experts have a large impact on the quantity and quality of care. In addition, cell phone cameras are the most ubiquitous optical sensors in the world (Breslauer et al., 2009; Rajchgot et al., 2017).

**Figure 12:**
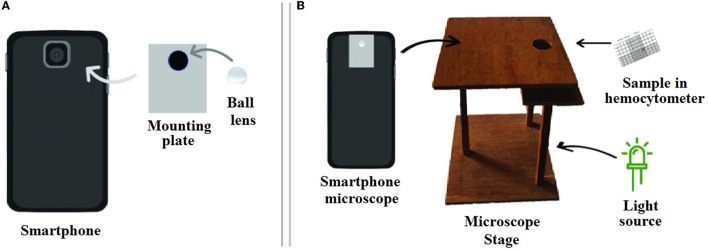
Schematic overview of a smartphone microscope setup. **(A)** Fabrication of ball lens onto mounting plate and the smartphone camera. **(B)** The set up of measurement system. Reproduced from Shrestha et al. (2020).

In the field of waterborne protozoa, Shrestha et al. (2020) developed a smartphone-based microscopic assay for the simultaneous detection of oocysts/cysts of *Cryptosporidium* and *G. lamblia* in vegetable and water samples. The device consisted of a ball lens 1 mm in diameter, an aluminum mounting plate to transform a smartphone, and a white LED as an illumination source. After concentration of oocysts and staining with Lugol's iodine, oocysts were counted using the smartphone microscope. In comparison to commercial bright field and fluorescence microscopes, the smartphone-based microscopic assay was a low-cost alternative for screening oocysts/cysts of *Cryptosporidium* spp., and *G. lamblia*. The LoD of *Giardia* ranged from 24 cysts/100 g for cucumber to 73 cysts/100 g for cabbage. The LoD for *Cryptosporidium* ranged from 11 oocysts/100 g for radish to 25 oocysts/100 g for cabbage, while the LoD of *Cryptosporidium* was lower than that of *Giardia* (Shrestha et al., 2020) (Figure 12).

**Figure 13:**
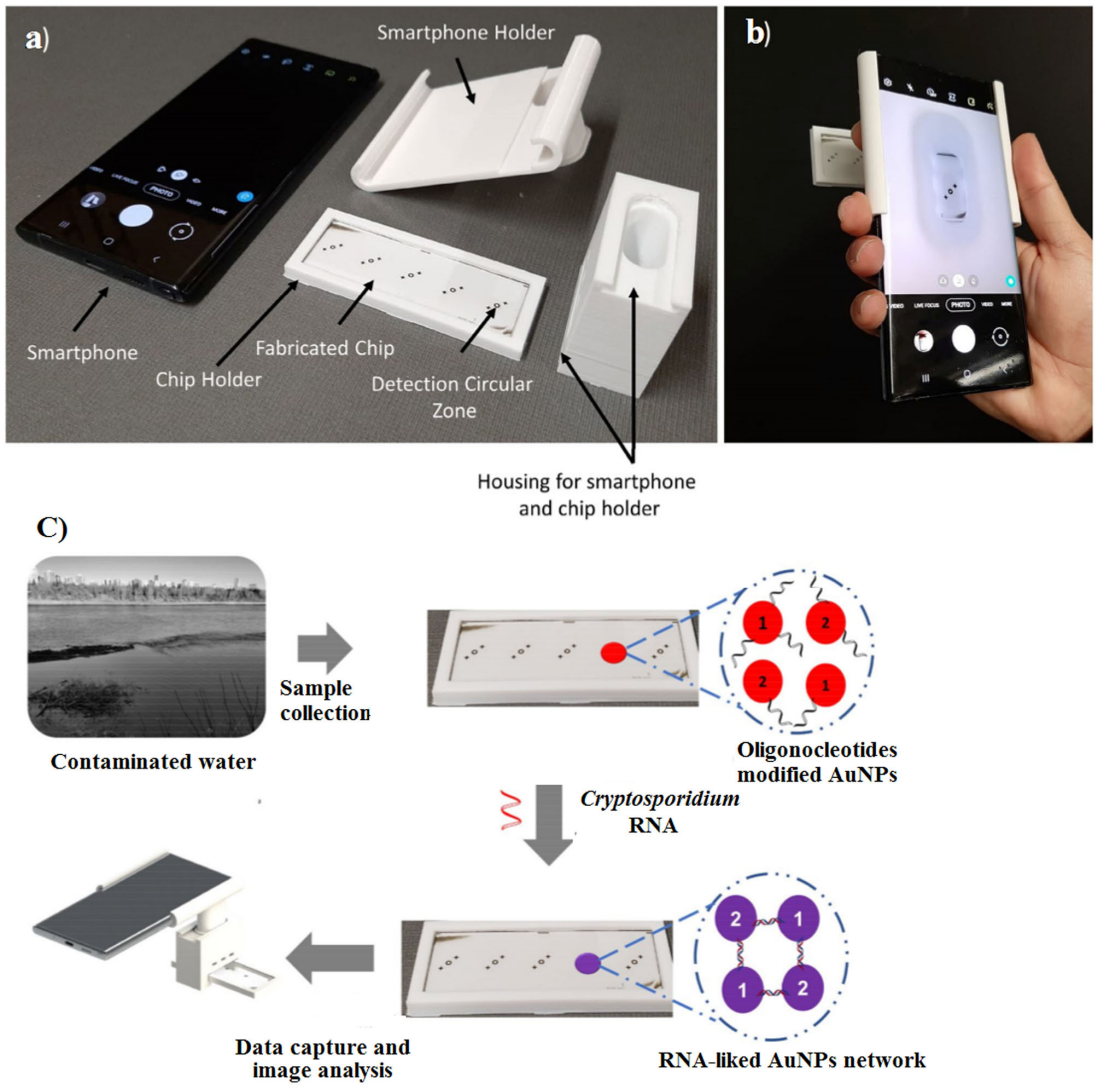
Fabricated chip and 3D portable holder assembly integrated with a smartphone. **(a)** The major components of the detection system, **(b)** the assembled detection system, and **(c)** schematic overview of sensing platform. Reproduced from Luka et al. (2021).

Recently, Luka et al. (2021) fabricated a 3D portable and smartphone-integrated on-chip colorimetric biosensor, which was invisible to the naked eye. In this regard, oligonucleotide-modified gold NPs (AuNPs) were used for the detection of *Cryptosporidium* RNA using UV–Vis spectroscopy. The color change of the AuNPs from red to blue after 5 min was an indicator for *Cryptosporidium* RNA. The advantages of these methods were the low sample volume (15 μL), short analysis time (~30 min), and high detection limit (5 μM) (Figure 13).

### 8.1. Future outlook

In recent years, advanced diagnostic procedures have been presented to overcome the limitations of available common techniques. Molecular biology assays, as gold standard methods, have been routinely utilized for rapid detection, identification, and differentiation of FWP. In addition, in recent decades, various studies have been conducted based on electrochemical and optical biosensors and nanobiosensors for the early detection of common waterborne pathogens. Although these methods frequently profit from good accuracy, reliability, and multiple sample processing, most of them suffer from the need for specialized expensive equipment, centralized services, infrastructure/or professional staff, and a lack of point-of-use (PoU) employment capabilities. The ASSURED criteria are an important guideline provided by the WHO for developing efficient POC devices to distinguish major human diseases (Syedmoradi et al., 2017; Ahmadi et al., 2020). Advances in digital health include mobile health, health information technology (IT), and wearable devices, and the acronym REASSURED (real-time connectivity, ease of specimen collection, affordable, sensitive, specific, user-friendly, rapid and robust, equipment-free or simple environmentally friendly, deliverable to end-users) has been offered for the development of diagnostic methods to address vital priorities such as global health emergencies (Land et al., 2019; Mahmoudi et al., 2022). Factors associated with the effective implementation of ASSURED diagnostic systems that should be considered in addressing POC diagnostic tests are real-time connectivity and ease of specimen collection (Land et al., 2019). Finally, for the successful diagnosis and management of infectious diseases, the necessity to fabricate smart biosensing systems is vital. We believe that smart biosensing platforms play an extremely significant role in diagnosing, as well as in predicting and controlling, future trends in infectious diseases, either epidemics or pandemics.

## Author contributions

HM and KO: conceived, designed, reviewing, and editing the manuscript. SN: data gathering, literature review, and writing the manuscript. FS: illustrations and graphics. All authors read and approved the final version of the manuscript.
